# Bromazolam Tablet Quantification and Analysis of Post‐Mortem Cases From the National Programme on Substance Use Mortality (NPSUM)

**DOI:** 10.1002/dta.70045

**Published:** 2026-02-24

**Authors:** Matthew Gardner, Molly F. Millea, Sam Craft, Rachael Andrews, Jennifer Scott, Stephen M. Husbands, Christopher R. Pudney, Oliver B. Sutcliffe, Caroline S. Copeland, Peter Sunderland

**Affiliations:** ^1^ Department of Life Sciences University of Bath Bath UK; ^2^ MANchester DRug Analysis & Knowledge Exchange (MANDRAKE), Department of Natural Sciences Manchester Metropolitan University Manchester UK; ^3^ Department of Psychology University of Bath Bath UK; ^4^ Centre for Academic Primary Care^,^ Bristol Medical School University of Bristol Bristol UK; ^5^ Centre for Pharmaceutical Medicine Research, Institute of Pharmaceutical Science King's College London London UK; ^6^ National Programme on Substance Use Mortality London UK

## Abstract

Bromazolam is a new psychoactive substance (NPS) benzodiazepine commonly identified by drug checking services and in post‐mortem toxicological analyses in the United Kingdom, Europe, and North America. At the time of writing, there are no studies that present quantitative analyses of bromazolam in street tablets. Here we describe the first quantitative analysis of bromazolam tablets, from samples submitted by UK drug checking services and police forces between 2022 and 2025. Using validated GC‐EI‐MS and ^1^H NMR methods, 47 tablet samples were quantified revealing a median bromazolam dose of 0.49 mg (interquartile range = 1.02 mg) per tablet, range of 0.09–5.4 mg. Over half of the tablet submissions (55%) mimicked the appearance of licensed pharmaceuticals alprazolam or diazepam, raising significant concerns around mis‐selling of street bromazolam tablets and the risks of unintentional high‐dose exposure to an NPS compound. To contextualise these findings, we also report post‐mortem data from the UK National Programme on Substance Use Mortality (NPSUM), in which bromazolam was detected in 396 drug‐related deaths between April 2021 and July 2024. Bromazolam detections in deaths rose from 28 deaths in 2021 to 160 deaths in 2023. Bromazolam was implicated in causing death in 82.8% of cases, with a median post‐mortem blood concentration of 43 ng/mL. Notably, bromazolam was co‐detected with an average of seven other substances per case, most commonly other central nervous system (CNS) depressants. These findings underscore the public health risks posed by bromazolam as an NPS benzodiazepine and highlight the urgent need for monitoring, harm reduction and forensic toxicology guidance.

## Graphical abstract

Tablets submitted to drug checking services that were found to contain bromazolam. Over half of these counterfeit tablets mimicked the appearance of licensed formulations (e.g., alprazolam Xanax bars and diazepam Roche 10 mg pills). Bromazolam is now the most commonly detected street benzodiazepine in drug poisoning deaths in the United Kingdom.

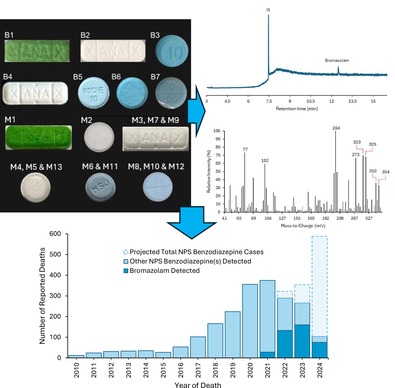

## 1 Introduction

Benzodiazepines are a large class of therapeutic drugs that act as central nervous system (CNS) depressants and are among the most widely prescribed drugs worldwide [1]. Benzodiazepines are licensed for a variety of conditions including severe acute anxiety, insomnia and acute muscle spasm and share a common mechanism as positive allosteric modulators of the GABA_A_ receptor [2, 3]. Illicit use of non‐prescribed benzodiazepines is common, where anxiolytic effects are often exploited to counteract the adverse effects of other substance use, including withdrawal symptoms [4]. In recent years, the number of deaths involving benzodiazepine use has increased in many parts of the world, most notably in the United States, Europe and Australia [5, 6, 7, 8]. Combining benzodiazepines with other sedatives such as opioids and/or alcohol puts users at increased risk of respiratory depression and death. Several studies have suggested that benzodiazepines may have played a role in as many as 80% of unintentional overdose deaths involving opioids [9, 10].

Traditionally, the illicit benzodiazepine market has been dominated by diverted prescription medications, typically confined to licensed drugs such as diazepam (Valium), temazepam (Restoril) and alprazolam (Xanax), among others [11]. However, as greater restrictions are placed on the prescribing of benzodiazepines, new and unlicensed compounds are emerging in global drug markets. Such drugs are referred to as new psychoactive substance (NPS) benzodiazepines, and, as of 2023, 36 of these compounds are being monitored by the European Monitoring centre for Drugs and Drug Addiction (EMCDDA) [6]. Bromazolam is a prominent example of an NPS benzodiazepine [6]. Unlike licensed compounds (e.g., diazepam/temazepam), NPS have not undergone clinical trials, and very little is known about their pharmacology or toxicity profiles [12]. Moreover, dosing and onset of action are unknown, and as production from illicit manufacturers is unregulated, there is likely to be significant variation in drug content within street benzodiazepine tablets [12, 13]. Risks associated with NPS benzodiazepines are therefore likely to be considerably higher than those from diverted medications, and, as such, they are increasingly being implicated in drug poisonings and deaths in several countries [6, 8]. Although initially sold under international non‐proprietary names, NPS benzodiazepines are now being used in the manufacture of falsified versions of traditional prescription benzodiazepines, increasing the risk of accidental exposure and unexpected effects [6]. These products are known as street benzodiazepines.

From its first emergence in the United States during 2016, bromazolam has now been detected in over 250 toxicology reports including both ante‐mortem and post‐mortem cases [14]. Bromazolam is now the most frequently detected street benzodiazepine in Scotland and by the Cardiff‐based drug checking service WEDINOS, often appearing in tablets mis‐sold as prescription benzodiazepines (e.g., Xanax) [15, 16]. At the time of writing, no quantitative analysis of bromazolam tablets is available in the literature. This constitutes an important evidence gap, as clinicians and public health organisations seek to deliver informed harm reduction messages and interventions. Characterising the marked variability in bromazolam concentrations will enhance clinical management and guide more targeted public health responses. Here we describe, to our knowledge, the first study to quantify bromazolam content in tablet samples, submitted by UK drug checking services and police forces. We also report UK post‐mortem data from the National Programme on Substance Use Mortality (NPSUM) involving bromazolam from April 2021 to July 2024.

## 2 Material and Methods

### 2.1 Sample Provenance

Bromazolam tablets from 20 individual batches/samples were analysed in this study, where quantitative analysis was performed on 47 bromazolam tablet samples in total. Samples were submitted to and analysed by the University of Bath and MANDRAKE (MANchester DRug Analysis and Knowledge Exchange), Manchester Metropolitan University. Thirteen individual batches of tablets were submitted to MANDRAKE. The tablets were homogenised and analysed to determine the mean concentration of the separate batches. Seven additional batches/samples were submitted to the University of Bath by Bristol Drugs Project, Devon and Cornwall police, Developing health and independence (DHI) and The Loop. Sample submissions are reported in Tables S1 and S2. Ten samples from batches B4, B5 and B6 submitted to the University of Bath for analysis were quantified to analyse intra‐batch variation of bromazolam dose. In total, 34 tablets were analysed at the University of Bath.

### 2.2 Chemicals and Reagents

Bromazolam reference standards were purchased from Cayman Chemical (University of Bath) and LGC Standards (MANDRAKE). Bromazolam‐d5 was purchased from Cayman Chemical, as 1 mg/mL in methanol. HPLC‐grade methanol, HPLC‐grade chloroform, chloroform‐d and DMSO‐d6 and methanol were purchased from Merck. All reference standards were stored in the dark at −20°C as per manufacturers' recommendations.

### 2.3 Sample Preparation and Screening

#### 2.3.1 GC‐EI‐MS Analysis (MANDRAKE)

A weight of homogenised powder (equivalent to a single tablet) was extracted/vortexed with HPLC‐grade methanol (2 mL) for 10 min and then filtered using a Whatman 0.45‐μm polyvinylidene difluoride (PVDF) syringe filter (Sigma‐Aldrich, Gillingham, UK). An aliquot (900 μL) of the filtrate was subsequently spiked with a solution of methyl stearate (100 μL, 700 μg/mL) to give the test solution containing the internal standard at 70 μg/mL.

#### 2.3.2 LC‐HR‐MS/MS Analysis (University of Bath)

One whole tablet was crushed and homogenised. Approximately 50 mg powder was extracted with 1 mL HPLC‐grade methanol (exact sample masses were weighed actuarially using a mass balance and used in subsequent quantification calculations). This solution was vortexed for 5 min and then centrifuged for 5 min at 12,000 rpm. An aliquot (100 μL) of the supernatant was diluted 1:1000 in HPLC methanol. 980 μL of this solution was spiked with bromazolam‐d5 (20 μL of 20 μg/mL) to give the test solution containing the internal standard at 200 ng/mL.

#### 2.3.3 ^1^H qNMR Analysis (University of Bath)

Tablets were weighed, crushed and extracted with chloroform. Solvent was removed from the organic extract, and the residue was quantified by ^1^H qNMR (Bath).

Additionally, IR and ^13^C NMR measurements were taken at the University of Bath for structural elucidation. All samples were screened for the presence of illicit drugs using LC‐HR‐MS/MS with the 2024 HighResNPS database (University of Bath).

### 2.4 Structural Elucidation

**FIGURE 1:**
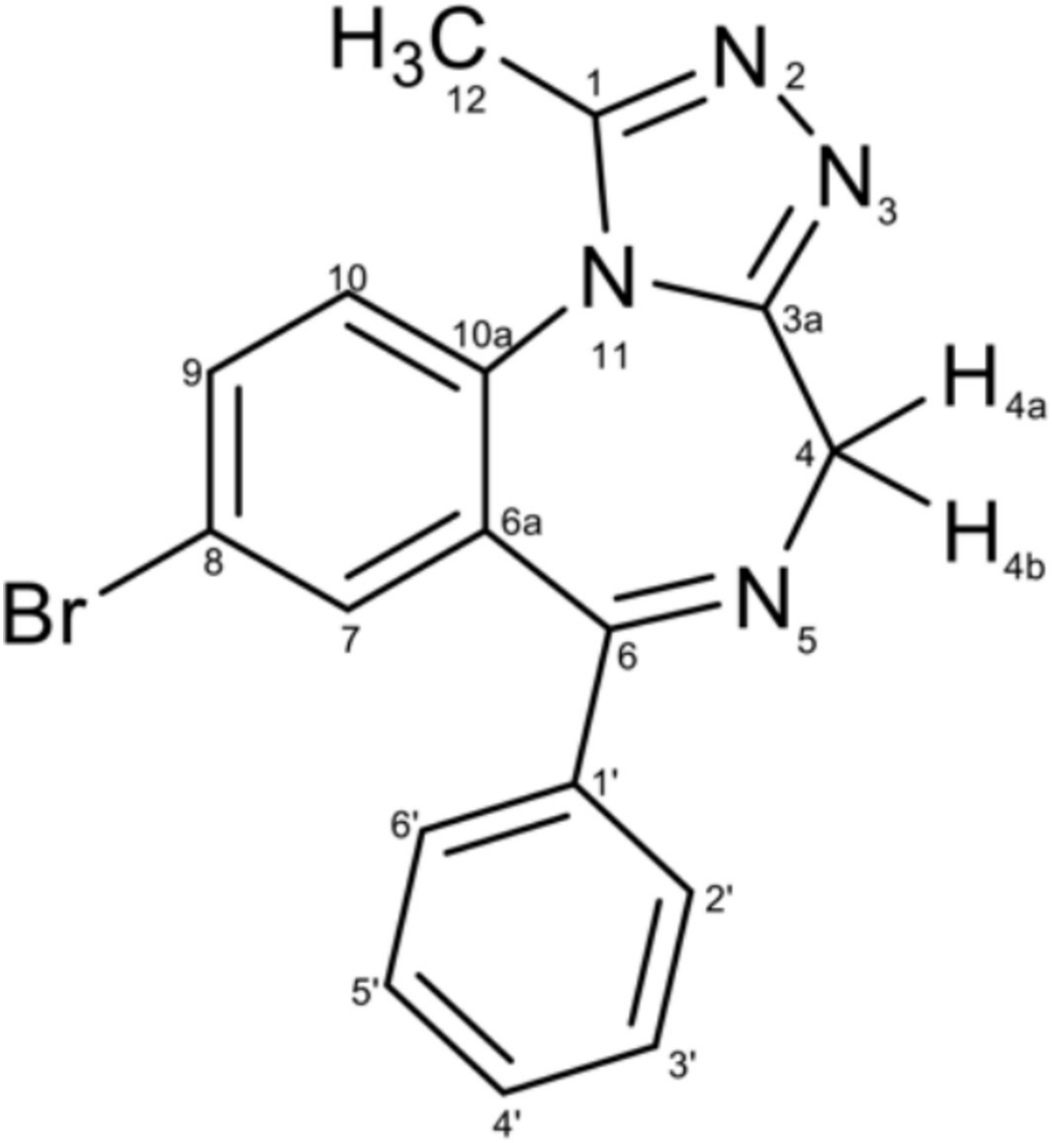
Chemical structure of bromazolam; numbering follows IUPAC convention.

Prior to quantitative analysis, structural elucidation of bromazolam was performed on tablet sample B1 (University of Bath) and is reported to aid future investigations. Infrared (IR) spectra were recorded on a PerkinElmer Spectrum 100 ATR‐FTIR spectrometer. Analysis was performed on a CHCl_3_ extract of bromazolam tablet sample B1 in a liquid cell. IR spectra for bromazolam tablet sample B1 are shown in Figure S1. NMR spectra were recorded in CDCl_3_ on 500 MHz spectrometers (Agilent ProPulse, Bruker AVANCE III). ^1^H and ^13^C NMR data were determined at 500 and 125 MHz, respectively. Chemical shifts are reported downfield from TMS. Coupling constants, *J*, are reported in Hz. Full structural elucidation was completed with 2D NMR correlation spectroscopy (COSY) (Figure S2), heteronuclear multiple bond correlation (HMBC) (Figure S3) and heteronuclear single quantum coherence (HSQC) (Figure S4). The measured ^1^H and ^13^C chemical shifts of bromazolam in CDCl_3_ are shown in Table S3, and numbering follows the structure presented in Figure 1.

### 2.5 ^1^H NMR Analysis

For bromazolam tablet samples submitted to the University of Bath, quantification was performed according to a previously validated ^1^H NMR method [17, 18]. Deuterated dimethyl sulfoxide (DMSO‐d6) was used as solvent and maleic acid as internal standard (IS), comparing the integrals of the IS alkene protons with the H7 and H9 integrals of bromazolam using TopSpin processing software. The following equation was used for the ^1^H qNMR quantitation:

$$\left[x\right]=\frac{n_{\mathrm{IC}}\cdot {\mathrm{Int}}_{x}\cdot {\mathrm{MW}}_{x}\cdot m_{\mathrm{IC}}}{n_{x}\cdot {\mathrm{Int}}_{\mathrm{IC}}\cdot {\mathrm{MW}}_{\mathrm{IC}}\cdot m_{s}}\cdot P_{\mathrm{IC}}$$

An exemplar labelled spectra of a bromazolam tablet extract (sample B4.1) is shown in Figure S5. Quantification results presented in Table 1 are given as mean (SD) for three repeats of the same sample.

**TABLE 1:** Quantification data for 47 bromazolam tablet samples seized 2022–2025. Batches B1‐B7 submitted to the University of Bath were quantified by ^1^H NMR. Ten tablets were analysed from batches B4, B5 and B6, tablet mass and bromazolam dose are reported as mean (SD). Batches M1–M13 submitted to MANDRAKE were quantified by GC‐EI‐MS.

Batch	Tablet mass (mg)	Bromazolam dose per tablet (mg)
B1	215.6	5.4 ± 0.03
B2	308.1	4.4 ± 0.05
B3	198.5	0.32 ± 0.02
B4	329.5 ± 3.2	1.95 ± 0.27
B5	162.2 ± 0.9	0.47 ± 0.08
B6	126.2 ± 3.0	0.50 ± 0.05
B7	228.7	0.09 ± 0.00
M1	330	1.10 ± 0.32
M2	173	0.46 ± 0.71
M3	312	2.52 ± 0.40
M4	234	1.04 ± 0.20
M5	211	1.38 ± 0.30
M6	84	0.48 ± 0.10
M7	314	0.20 ± 0.04
M8	126	0.20 ± 0.01
M9	318	1.69 ± 0.10
M10	129	0.36 ± 0.02
M11	86	0.48 ± 0.10
M12	124	1.38 ± 0.30
M13	225	0.46 ± 0.71

### 2.6 Gas Chromatography–Electron Ion–Mass Spectrometry (GC‐EI‐MS)

Quantification of bromazolam tablet samples submitted to MANDRAKE was performed according to a previously validated gas chromatography–electron ion–mass spectrometry (GC‐EI‐MS) method [19]. GC‐EI‐MS analysis was performed using an Agilent 7890B GC (Agilent Technologies, Wokingham, UK) as previously described. All standards and test samples were prepared as solutions in HPLC‐grade methanol with no derivatisation. Methyl stearate (70 μg/mL) was used as an internal standard, and each standard/test sample was injected in triplicate. *Qualitative analysis*: Mass spectra were obtained in full scan mode (*m*/*z* = 40–550) for bromazolam (t_R_ = 12.48 min) and methyl stearate (t_R_ = 7.45 min) respectively. *Quantitative analysis*: Obtained in selective ion monitoring (SIM) mode using three specific fragment ions for bromazolam (*m/z* = 325, 352 and 353) and methyl stearate (*m/z* = 74.0, 87.0 and 143.0) respectively. The spectral data were compared to a reference standard of bromazolam, and this is presented in Figure S6. GC‐MS method validation data is shown in Table S4. The mean dose for the three replicates of each sample was calculated along with the relative standard deviation (% RSD) expressed as an upper/lower limit of the mean.

### 2.7 LC‐HR‐MS/MS Quantitative Analysis

Quantification of bromazolam tablet samples from batches B4‐B7 submitted to Bath was performed using quantitative LC‐HR‐MS/MS analysis to confirm the accuracy of our previously validated ^1^H NMR quantification approach. LC‐HR‐MS/MS analyses were performed using an Agilent QToF 6545 setup as previously reported [20]. All reference standards and test samples were prepared as solutions in HPLC‐grade methanol with no derivatisation. Bromazolam‐d5 (200 ng/mL final concentration) was used as an internal standard and each standard/test sample was injected in triplicate. *Qualitative analysis*: Mass spectra for bromazolam (t_R_ = 2.250 min) and bromazolam‐d5 (t_R_ = 2.25 min) were obtained in all‐ions mode with three collision energy scan segments at 0, 20, and 40 eV. *Quantitative analysis*: Quantification was performed by isotopic dilution mass spectrometry (IDMS), previously validated for designer benzo quantification [21]. The MS was operated in a targeted MRM mode with two collision energy scan (CID) segments at 0 and 40 eV. Three specific ions for bromazolam (*m/z* = 353.0365, 325.0214 and 274.1211) and bromazolam‐d5 (*m/z* = 358.0689, 210.1073 and 279.1512) were used (Table S5). The six‐point calibration curve is shown in Figure S7, where an *r* ^2^ value of 0.999 was achieved. We used bromazolam reference standard at concentrations 0, 100, 200, 300, 400 and 500 ng/mL, where each of these were spiked with 200 ng/mL bromazolam‐d5. For each sample (tablet extract), 980 μL of this solution was spiked with 20 μL of 20 μg/mL bromazolam‐d5 and then ran immediately. Quantification results were processed in Agilent's MassHunter Q‐TOF Quant‐My‐Way software. The mean dose for the three replicates of each sample was calculated along with the relative standard deviation (% RSD) expressed as an upper/lower limit of the mean.

### 2.8 Post‐Mortem Data and the NPSUM

NPSUM regularly receives voluntary reports from coroners in England, Wales and Northern Ireland on deaths related to psychoactive drug use. A death is referred to a coroner if it has an unknown cause; is violent or unnatural; sudden and unexplained; occurred during an operation or before the person came out of an anaesthetic; or potentially caused by an industrial disease or poisoning [22]. Toxicology tests are requested dependent upon individual case circumstances at the discretion of the coroner and consulting pathologist. The King's College London Biomedical & Health Sciences, Dentistry, Medicine and Natural & Mathematical Sciences Research Ethics Subcommittee reconfirmed (August 2025) that NPSUM does not require research ethics committee review as all subjects are deceased. A retrospective study design identified all cases with NPS benzodiazepine detections, including those with bromazolam, from the NPSUM by searching the entire NPSUM database (records received 1997 to 1 November 2024) in the post‐mortem drug fields for the numerical codes assigned to the NPS benzodiazepines. The projection for expected post‐mortem cases was calculated by multiplying the number of reported cases by the data cut date by the average increase in cases when complete reporting is received for the year. Circumstances that lead to death are categorised on the Record of Inquest issued by the coroner as follows:

Cause 1a: The immediate cause of death (and underlying if no 1b or 1c cited)

Cause 1b: Any disease/circumstances underlying Cause 1a

Cause 1c: Any disease/circumstance underlying Cause 1b

Cause 2: Any disease/circumstance that did not cause the death but contributed in some way

It is not a requirement for a Cause 1b, 1c or 2 to be cited for all deaths. Underlying cause of death was identified using these criteria. Implication of bromazolam in a death indicates that it was identified by the coroner and/or consulting pathologist as a causal factor following their investigations and listed as such as a cause of death on the Record of Inquest.

## 3 Results and Discussion

### 3.1 Visual Analysis

**FIGURE 2:**
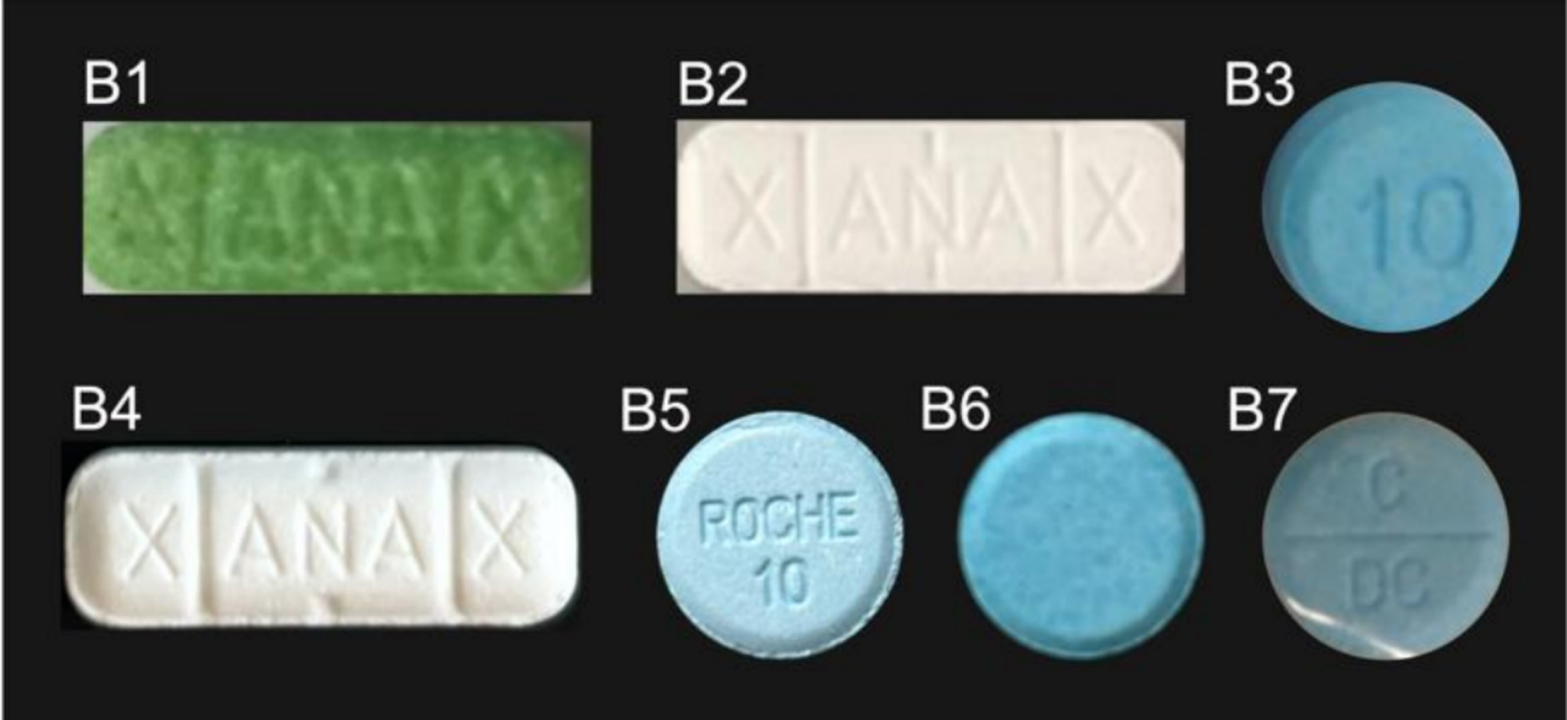
Appearance of bromazolam tablets from batches B1‐B7 submitted to University of Bath 2023–2025.

Appearance of tablets submitted to the University of Bath (batches B1‐B7) is shown in Figure 2 and described in Table S1. Tablets from four of seven batches were direct imitations of licenced pharmaceutical benzodiazepines alprazolam (‘Xanax’, B1, B2 and B4) or diazepam (as ‘Roche 10 mg’, B5). Appearance of tablets submitted to MANDRAKE (batches M1–M13) is shown in Figure S8 and described in Table S6. Tablets from seven of 13 batches were direct imitations of alprazolam (‘Xanax’, M1, M3, M7 and M9) or diazepam (‘MSJ’, M6, M11 and ‘Bensedin’, M2). The remaining exhibits comprised blue (M8, M10 and M12) or white (M4, M5 and M13) tablets that bore no identifying markings.

### 3.2 Quantitative Analysis

Quantitative analysis of 47 bromazolam tablets submitted to the University of Bath and MANDARKE is reported in Table 1. Median bromazolam dose per tablet was 0.49 mg (IQR = 1.02 mg), with a range of 0.09–5.4 mg. Median tablet mass was 213.3 mg (IQR = 180.8 mg), with a range of 84–330 mg. Tablets submitted to MANDRAKE were analysed by a previously validated GC‐EI‐MS approach. Quantitative analysis revealed a median bromazolam dose of 0.48 mg (IQR = 0.92 mg) per tablet sample. Median tablet mass was 211 mg (range = 86–330 mg).

Bromazolam tablets submitted to the University of Bath were analysed by a previously validated ^1^H qNMR method. Quantitative analysis revealed a median bromazolam dose per tablet 0.50 mg (IQR = 2.78 mg) across 34 tablets. Median tablet mass was 215.6 mg (range = 126.2–329.5 mg). Intra‐batch variance was assessed for batches B4, B5 and B6 by analysing 10 tablets with ^1^H qNMR. Bromazolam dose per tablet ranged from 1.66 to 2.41 mg in batch B4, 0.28 to 0.57 mg in B5 and 0.41 to 0.59 mg in B6 (Table S2). To confirm the accuracy of our previously validated ^1^H NMR approach, we compared quantification results to an LC‐HR‐MS/MS method (Table S7). Cocaine was detected as a minor constituent in tablet sample B7 by LC‐HR‐MS/MS. Transition reactions used in LC‐HR‐MS/MS detection of cocaine are reported in Table S8.

### 3.3 .Post‐Mortem Bromazolam Data

Between the reporting of the first death in April 2021 and the last captured by the data cut date in July 2024, a total of 396 deaths occurred in England, Wales and Northern Ireland in which bromazolam was detected during post‐mortem sample toxicological analysis and were reported to the NPSUM. The toxicology reports detailing these detections originated from 10 different toxicology laboratories (six NHS hospital toxicology laboratories and four private forensic toxicology companies). Blood concentration levels were available for 299 (75.5%) of these cases (though in five cases ante‐mortem concentrations are reported). Blood concentrations varied between 0.09 and 1100 ng/mL, and the median concentration was 43 ng/mL.

Other available case information is reported in Table 2. Briefly, the majority of cases were male (78.8%; *n* = 312) and ages ranged between 16 to 68 (mean [±SD] = 39.8 [+10.0]). Where index of multiple deprivation could be calculated (90.4% of cases, *n* = 358/396), over half (54.1%) were registered as either of no fixed abode (NFA; 4.8%, *n* = 17/358) or to a home address within a location classified as one of the most deprived areas in the United Kingdom (i.e., Quintile 1 of the IMD index; 49.3%, *n* = 176/358). Bromazolam specifically was implicated in causing death in 82.8% of cases (*n* = 328/396), and in the majority of cases (94.7%; *n* = 375/396), acute drug/alcohol toxicity was cited as the underlying cause of death.

**TABLE 2:** Case information for the 396 deaths involving bromazolam reported to the NPSUM in the United Kingdom.

Age	Mean (±SD): 39.8 (±10.0)
Gender	
Male	78.8% (*n* = 312)
Female	21.2% (*n* = 84)
IMD quintile^a^, ^b^	
NFA	4.8% (*n* = 17)
1	49.3% (*n* = 176)
2	21.3% (*n* = 76)
3	12.6% (*n* = 45)
4	7.3% (*n* = 26)
5	5.0% (*n* = 18)
Bromazolam blood concentration	Median (IQR): 43 ng/mL (78 ng/mL)
Bromazolam implicated in death	
Yes	82.8% (*n* = 328)
No	17.2% (*n* = 10)
Underlying Cause of death	
Acute drug/alcohol toxicity	94.7% (*n* = 375)
Respiratory depression/failure	2.0% (*n* = 8)
Cardiac disease	1.5% (*n* = 6)
Other	1.8% (*n* = 7)
Manner of death	
Accidental (drug‐related)	96.5% (*n* = 382)
Natural causes combined with drug use	2.0% (*n* = 8)
Suicide	0.8% (*n* = 3)
Undetermined	0.8% (*n* = 3)
Total number of drugs/medications detected	Mean (±SD): 7.0 (±2.9)

^a^ Data available for 358 decedents (90.4%)—37 in Wales and IMD calculator offline at time of writing; 1 living in an area with a new postcode not mapped by the 2019 IMD England calculator launch.

^b^ Valid %.

**FIGURE 3:**
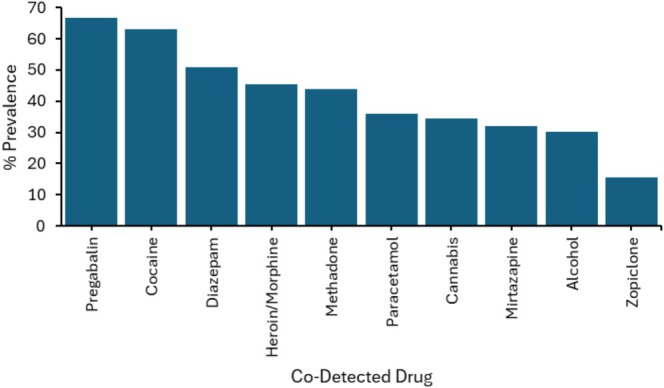
Drugs/medications most commonly co‐detected with bromazolam during post‐mortem analysis.

In addition to bromazolam, at least one other illicit drug/prescribed medication was detected in all cases. The mean (±SD) number of total drugs/medications detected was 7.0 (±2.9), and the most frequently detected were pregabalin (66.7%, *n* = 264), cocaine (62.9%, *n* = 249) and diazepam (51.0%, *n* = 202; see Figure 3 for the prevalence of additional drugs/medications detected across all cases). Notably, at least one other benzodiazepine was also detected in 64.4% (*n* = 255) of cases; most common were diazepam (51.0%, *n* = 202), clonazepam (9.1%, *n* = 36) and alprazolam (8.3%, *n* = 33). Additionally, the number of reported deaths where bromazolam was detected in post‐mortem analysis has been consistently increasing from 2020 to 2024, with bromazolam detected in 7.5% of total deaths with NPS benzodiazepine detections in 2021 (*n* = 28/375), rising to 60.2% (*n* = 160/266) and 73.5% (*n* = 75/102) in 2023 and 2024, respectively (Figure S9).

## 4 Discussion

NPS benzodiazepines are illegal in the United Kingdom and carry greater risks from consumption than licensed pharmaceuticals diverted from prescription (e.g., diazepam). Risk of accidental overdose is heightened as dosage may be highly variable between batches [6]. NPS have also not been examined in clinical trials, meaning that the pharmacokinetics, pharmacodynamics and safety profile of these compounds are unknown [12]. Bromazolam is among the most prevalent NPS benzodiazepines, routinely reported by drug checking services (e.g., WEDINOS) and in post‐mortem analyses in the United Kingdom, the United States and Canada [16, 23, 24, 25, 26]. Here, we present, to our knowledge, the first study to quantify bromazolam content in whole tablets, submitted to the University of Bath and MANDRAKE by drug checking services and UK police forces, 2022–2025. We further show NPSUM data that tracks the involvement of bromazolam in deaths following drug use in the United Kingdom from April 2021 to July 2024.

From the 20 batches of tablet submissions analysed in this study, 11 (55%) presented as direct imitations of licenced benzodiazepines sold by global pharmaceutical companies. These were copies of either alprazolam (seven total) or diazepam (total total) products, likely produced with the intent to trick customers into believing they had purchased a pharmaceutical product. Mis‐selling of street benzodiazepine tablets as licenced pharmaceuticals is problematic as users may be under the impression they are consuming a clinically approved compound at a known dose; however, they may instead be consuming an NPS with an unknown toxicity profile, present at an unknown quantity. Concerningly, this practice is highly prevalent among drug dealers in the UK; diazepam was identified in only 52% (745) of 1408 submissions to the UK drug checking service WEDINOS between April 2023 to March 2024 where it was the purchase intent, with bromazolam identified in 34% (482) of instances [16].

Bromazolam tablets are manufactured in clandestine laboratories, meaning doses are unregulated and potentially highly variable. This makes it difficult for users to judge quantities to consume for the desired effect, thereby increasing the risk of negative outcomes. In samples submitted to the University of Bath and MANDRAKE across 2022–2025, quantitative analysis revealed a median bromazolam dose of 0.49 mg (IQR = 1.02 mg) per tablet, with a range of 0.09–5.4 mg. This range spans over the three dosage brackets (light: 0.5–1 mg, common: 1–2 mg and strong: 2–4 mg) reported for bromazolam on a popular online harm reduction website (TripSit) [27], highlighting the marked variability and consequent unpredictability of risk in street benzodiazepine use. Bromazolam dosing must be extracted from sources of self‐reporting due to a current lack of activity studies. Light and common doses (0.5–2 mg) are lower than that of diazepam and closer to alprazolam [28]. Given the classification of alprazolam as a high‐potency benzodiazepine, these self‐reported data therefore indicate an intermediate to high potency for bromazolam; however, this is yet to be experimentally determined [28]. The purported dosing of bromazolam falling closer to that of alprazolam than diazepam is, however, consistent with the ability of triazolobenzodiazepine derivatives to tend towards a higher activity in potentiating GABA_A_ receptor affinity [29].

Samples B1 and B2 contained 5.4 and 4.4 mg of bromazolam per tablet, higher than the strongest dosage reported on Tripsit (4 mg). Consumption of these tablets may present the user a significant risk of an acute benzodiazepine overdose, where adverse effects cited for bromazolam include irregular heartbeat, memory loss and loss of consciousness [27]. These tablets also presented as ‘Xanax’ imitations where, in total, 7/20 batches of tablets analysed in this study had the same appearance. Quantitative analysis identified a range of 0.2–5.4 mg bromazolam in these tablets (median = 1.95 mg). This variation in dose found within tablets that were likely sold as the same product further underscores the risk of street benzodiazepine use; consumers anticipating a repeat dose may be caught off guard by a tablet containing over 20× the dose previously encountered. This is particularly dangerous should the user be engaged in polydrug use. Similarly, we also identified significant intra‐batch variation in batch B4, where the 10 tablets analysed were found to contain a range of 1.66–2.41 mg bromazolam. Moreover, cocaine was detected at a trace level in sample B7.1; however, it was not present in sufficient quantity for quantification by our ^1^H NMR approach. This dose is hence not likely to be pharmacologically relevant; however, it does further highlight the lack of quality control associated with the production of illicit street benzodiazepine tablets.

Bromazolam was detected in post‐mortem analysis of 396 deaths reported to the NPSUM in the United Kingdom between April 2021 and November 2024, where bromazolam was implicated in causing death in 328 (82.8%) cases. The risks associated with bromazolam use should therefore be well recognised once the identity of this substance is known to the user. Notably, in 382 of 396 (96.5%) cases where bromazolam was identified, the manner of drug‐related death was noted as accidental, indicating some fatal use of bromazolam may have occurred though unintentional consumption. Users may have thought they were consuming a clinically approved benzodiazepine, present at a known dose that was hence safer to combine with other sedative substances and/or alcohol. This is evidenced by the high proportion of cases where another benzodiazepine was co‐detected alongside bromazolam (64.4%). NPSUM data further show that the demographic of people who died following consumption of bromazolam is similar to that of others who died following other NPS use since the introduction of the Psychoactive Substance Act (2016) and thus further indicates that this legislation has driven NPS use in a deprived demographic who experience heightened risk of drug‐related harms [30].

Given the predicted intermediate to high potency of bromazolam [28], its use in combination with other CNS depressants is likely to be particularly unsafe as these drugs can increase benzodiazepine toxicity, where respiratory depression, coma and death are possible outcomes [23, 27]. Indeed, NPSUM data show for the 396 deaths involving bromazolam, a mean average of 7 (±2.9) drugs/medications were co‐detected, where among the most prevalent were the CNS depressants pregabalin (66.7% of cases), diazepam, heroin and methadone. One study that analysed post‐mortem blood concentrations of bromazolam from 96 cases in England and Wales 2021–2022 also concluded that death was most frequently associated with polydrug use, including CNS depressants, where pregabalin was again co‐detected in 66% of cases [23]. Indeed, there will be some overlap between cases in the NPSUM dataset and those reported by Hikin et al. [23] as this laboratory undertakes post‐mortem casework for several coroners across England and Wales. However, it is not possible to determine which individual cases overlap, as not all coroners served by this laboratory report to the NPSUM. The dataset presented in this study included 299 cases with quantified bromazolam concentrations, compared with 96 reported by Hikin et al. Although median concentrations in the NPSUM data (43 ng/mL, range = 0.09–1100 ng/mL) were somewhat lower but more variable than the mean reported by Hikin et al. (64.6 ng/mL, range = < 1–425 ng/mL), both studies consistently identified bromazolam predominantly in the context of polydrug use rather than in isolation. Bromazolam toxicity in isolation is therefore rarely the cause of drug‐related death in post‐mortem analyses where the compound is detected. A study from British Columba, Canada reported 41 detections of bromazolam in post‐mortem analyses from 2021, where the cause of death in all instances was identified as mixed drug (illicit/prescription) toxicity [24]. Another study identified bromazolam in 112 post‐mortem cases in Texas, USA, from 2021 to 2023, where polydrug use was identified in 99% of deaths [25]. Another study in from Indiana published similar findings from 94 cases during 2023, where bromazolam was never detected in isolation [26]. NPSUM data revealed a median blood bromazolam concentration of 43 ng/mL for the 396 cases analysed in this study. This is consistent with previous studies in both the United Kingdom and the United States where polydrug use in acute drug/alcohol toxicity was identified as the manner of death [23, 25]. It is evident from the NPSUM post‐mortem data that bromazolam is increasingly becoming a widely used NPS benzodiazepine in the United Kingdom, which contributes to drug‐related deaths through acute drug/alcohol toxicity, respiratory depression and suicide. NPSUM data further show that deaths involving bromazolam increased consistently from 2021 to 2023, with an expected further increase in 2024 (full data not yet reported), highlighting both the increasing prevalence of bromazolam tablets and the need for testing, monitoring and care.

## 5 Conclusion

The goal of this study was to quantify the bromazolam content in tablets submitted by UK drug checking services and police forces to the University of Bath and MANDRAKE and to analyse post‐mortem cases involving bromazolam reported to the NPSUM. To our knowledge, this is the first study to quantify the bromazolam content from street benzodiazepine tablets anywhere in the world. We show 11 of 20 batches (55%) of tablets analysed were imitations of pharmaceutical products diazepam or alprazolam, likely mis‐sold with the intent to make customers believe they were purchasing licensed medications. We report significant variation in bromazolam content across a total of 47 tablet samples, and the presence of cocaine identified in one sample, highlighting the well‐known lack of quality control exercised in the manufacture of street benzodiazepines and the heightened risk of consumption. We also report significant variation in bromazolam content across tablets with similar appearance, in addition to inter‐batch variation between tablets analysed as part of the same submission, where users anticipating a repeat dose may be at risk of acute benzodiazepine overdose. Quantification of the submitted tablets reported in this paper gives indication as to the potential doses of bromazolam that are being consumed illicitly in the United Kingdom. Understanding the marked variability in bromazolam content has important implications for toxicology interpretations and the design of harm reduction initiatives. In post‐mortem and clinical toxicology, quantification data can assist in the interpretation of detected blood concentrations to distinguish between low range exposure, non‐fatal overdoses and fatal poisonings. Regular updates regarding emerging NPS are therefore needed, with the incorporation of these compounds into toxicology panels. From a public health perspective, communicating the unpredictability of bromazolam content can inform clinical practice for the treatment of overdoses and also harm reduction messaging to guide targeted outreach to at‐risk populations. We note however that this study has taken advantage of convenience sampling and that analysis performed on a larger sample size of seized/submitted bromazolam tablets is required to provide a clearer picture of dosage per tablet in the United Kingdom and beyond. Post‐mortem data from the NPSUM highlight danger of active polydrug use involving bromazolam, where the potential to cause harm is greatest when this NPS is combined with other CNS depressants including opioids, alcohol and benzodiazepines. Presentation of NPSUM data should aid interpretation of post‐mortem bromazolam blood concentrations when detected alone or when co‐detected with other substances in cases of drug‐related death.

## Funding

The authors have nothing to report.

## Conflicts of Interest

The authors declare no conflicts of interest.

## Supporting information


**Table S1:** Appearance of bromazolam tablets from seven batches submitted to the University of Bath for analysis.
**Table S2:** dta70045‐sup‐0001‐SuppInfo.docx. ^1^H NMR quantification of bromazolam tablet samples from batches B4, B5 and B6 submitted to the University of Bath. Ten samples were analysed for each batch to show intra‐batch variance. NMR quantitative analyses are given as a mean ± SD for three repeat experiments of the same sample. Mean values are also given for tablet mass and bromazolam dose across individual batches.
**Table S3:** dta70045‐sup‐0001‐SuppInfo.docx. ^1^H and ^13^C NMR assignments of bromazolam. ^a^Chemical shifts (in ppm) were determined with reference to TMS. ^b^Spectra determined at 500 MHz. ^c^Spectra determined at 126 MHz. ^d,e^Equivalent environments with same chemical shift.
**Table S4:** GC‐MS validation data for the quantification of bromazolam.
**Table S5:** Transition reactions monitored in LC‐HR‐MS/MS quantification of bromazolam.
**Table S6:** Appearance of bromazolam tablets from 13 batches submitted to MANDRAKE for analysis.
**Table S7:**. Comparison of ^1^H NMR Bromazolam quantification method using isotopic dilution LC‐MS/MS analysis. LC‐MS/MS quantitative analyses represent mean ± SD of three injections of the same sample (tablet extract). ^1^H NMR quantitative analyses represent three scans of same sample (tablet extract).
**Table S8:**. Transition reactions monitored in LC‐HR‐MS/MS detection of cocaine from sample B7.
**Figure S1:**. IR spectrum of bromazolam sample B1 (tablet extract in CHCl_3_) measured in liquid cell. IR vmax (KBr, cm^−1^) 3401, 2923 Ar(C‐H) stretch, 1758 Ar(C=N) stretch, 1485 Ar(C=C) stretch, 1184 (C‐N) stretch, 733 Ar(C‐H) bend, 698 (C‐Br) stretch.
**Figure S2:**. Homonuclear Correlation Spectroscopy (COSY) analysis of bromazolam tablet sample B1. Acquired in CDCl_3_.
**Figure S3:**. Heteronuclear Multiple Bond Correlation (HMBC) analysis of bromazolam tablet sample B1. Acquired in CDCl_3_.
**Figure S4:** Heteronuclear Single Quantum Coherence (HSQC) spectrum of bromazolam tablet extract from sample B1. Acquired in CDCl_3_.
**Figure S5:**. Exemplar ^1^H NMR spectrum of bromazolam tablet extract B4.1. Ran in deuterated DMSO‐D6 (singlet peak at 2.5 ppm). Maleic acid used as internal standard showing singlet peak at 6.27 ppm. H7 and H9 integrals of bromazolam used in quantification are shown at 7.79 ppm, doublet (d) and 7.98 ppm, double of doublets (dd).
**Figure S6:** (a) Representative total ion chromatogram of bromazolam (1 mg/mL) containing methyl stearate (IS, 70 μg/mL) in methanol. (b) EI‐MS spectrum (+ve ion mode) of bromazolam (tR = 12.48 min) standard. (c) Representative total ion chromatogram of Green “XANAX” embossed bar (Sample M1) containing methyl stearate (IS, 70 μg/mL). (d) EI‐MS spectrum (+ve ion mode) of Sample M1 (tR = 12.48 min).
**Figure S7:**. LC‐HR‐MS/MS isotopic dilution calibration curve for bromazolam/bromazoalm‐d5.
**Figure S8:** Appearance of bromazolam tablets from batches M1–M13 submitted to MANDRAKE 2022–2025.
**Figure S9:** Total number of deaths with bromazolam or other NPS benzodiazepine detections reported to the NPSUM by 1 November 2024. *Note:* Due to the time taken between a death occurring and conclusion of coronial inquest, which is when cases are reported to the NPSUM, it is anticipated that further cases will be reported where death occurred 2022–2024. The projected number of deaths anticipated to be received has been calculated using previous jurisdiction reporting trends.

## Data Availability

The data that support the findings of this study are available from the corresponding author upon reasonable request.
