# Supplementary material for: Bromazolam Tablet Quantification and Analysis of Post‐Mortem Cases From the National Programme on Substance Use Mortality (NPSUM)
Source: Drug Test Anal. 2026 Feb 24;18(4):543–51. doi: 10.1002/dta.70045 (PMC13040413; doi:10.1002/dta.70045)
Supplement: Supplementary file 1 — Table S1: Appearance of bromazolam tablets from seven batches submitted to the University of Bath for analysis. Table S2: dta70045‐sup‐0001‐SuppInfo.docx. 1H NMR quantification of bromazolam tablet samples from batches B4, B5 and B6 submitted to the University of Bath. Ten samples were analysed for each batch to show intra‐batch variance. NMR quantitative analyses are given as a mean ± SD for three repeat experiments of the same sample. Mean values are also given for tablet mass and bromazolam dose across individual batches. Table S3: dta70045‐sup‐0001‐SuppInfo.docx. 1H and 13C NMR assignments of bromazolam. aChemical shifts (in ppm) were determined with reference to TMS. bSpectra determined at 500 MHz. cSpectra determined at 126 MHz. d,eEquivalent environments with same chemical shift. Table S4: GC‐MS validation data for the quantification of bromazolam. Table S5: Transition reactions monitored in LC‐HR‐MS/MS quantification of bromazolam. Table S6: Appearance of bromazolam tablets from 13 batches submitted to MANDRAKE for analysis. Table S7:. Comparison of 1H NMR Bromazolam quantification method using isotopic dilution LC‐MS/MS analysis. LC‐MS/MS quantitative analyses represent mean ± SD of three injections of the same sample (tablet extract). 1H NMR quantitative analyses represent three scans of same sample (tablet extract). Table S8:. Transition reactions monitored in LC‐HR‐MS/MS detection of cocaine from sample B7. Figure S1:. IR spectrum of bromazolam sample B1 (tablet extract in CHCl3) measured in liquid cell. IR vmax (KBr, cm−1) 3401, 2923 Ar(C‐H) stretch, 1758 Ar(C=N) stretch, 1485 Ar(C=C) stretch, 1184 (C‐N) stretch, 733 Ar(C‐H) bend, 698 (C‐Br) stretch. Figure S2:. Homonuclear Correlation Spectroscopy (COSY) analysis of bromazolam tablet sample B1. Acquired in CDCl3. Figure S3:. Heteronuclear Multiple Bond Correlation (HMBC) analysis of bromazolam tablet sample B1. Acquired in CDCl3. Figure S4: Heteronuclear Single Quantum Coherence (HSQC) spectrum of brom [file DTA-18-543-s001.docx]

**Supplementary Information**

**Bromazolam tablet quantification and analysis of post-mortem cases from the National Programme on Substance Use Mortality (NPSUM)**

Matthew Gardner,^1^ Molly F. Millea,^2^ Sam Craft,^3^ Rachael Andrews,^1^ Jennifer Scott,^4^ Stephen M. Husbands,^1^ Christopher R. Pudney,^1^ Oliver B. Sutcliffe,^2^* Caroline S. Copeland, ^5,6^* Peter Sunderland^1^*

^1^Department of Life Sciences, University of Bath, Bath, UK

^2^MANchester DRug Analysis & Knowledge Exchange (MANDRAKE), Department of Natural Sciences, Manchester Metropolitan University, Manchester, UK

^3^Department of Psychology, University of Bath, Bath, UK

^4^Centre for Academic Primary Care^,^ Bristol Medical School, University of Bristol, Bristol, UK

^5^Centre for Pharmaceutical Medicine Research, Institute of Pharmaceutical Science, King’s College London, London, UK

^6^National Programme on Substance Use Mortality, London, UK

**Table S1.** Appearance of bromazolam tablets from 7 batches submitted to the University of Bath for analysis.

| **Batch** | **Date of submission** | **Source** | **Tablet appearance** |
| --- | --- | --- | --- |
| B1 | March 2023 | BDP | 1 x Green ‘xannax’ embossed bars |
| B2 | May 2023 | BDP | 1 x White ‘xannax’ embossed bars |
| B3 | May 2024 | Avon and Somerset police | 1 x Blue ‘10’ embossed |
| B4 | August 2024 | Devon and Cornwall police | 36 x White ‘xannax’ embossed bars |
| B5 | November 2024 | The Loop | 20 x Blue ‘Roche 10’ embossed circular tablets |
| B6 | January 2025 | Devon and Cornwall police | 89 x Blue, circular tablets, no markings |
| B7 | March 2025 | DHI | 1 x Blue ‘C/DC’ embossed |

**Table S2**. ^1^H NMR quantification of Bromazolam tablet samples from batches B4, B5 and B6 submitted to the University of Bath. Ten samples were analysed for each batch to show intra-batch variance. NMR quantitative analyses are given as a mean ± SD for three repeat experiments of the same sample. Mean values are also given for tablet mass and bromazolam dose across individual batches.

| **Batch** | **Sample** | **Tablet mass (mg)** | **Quantitative analysis (mg/tablet)** |
| --- | --- | --- | --- |
| B4 | 4.1 | 327.8 | 1.96 ± 0.06 |
|  | 4.2 | 326.4 | 1.97 ± 0.03 |
|  | 4.3 | 335.2 | 2.27 ± 0.02 |
|  | 4.4 | 333.1 | 2.32 ± 0.06 |
|  | 4.5 | 330.0 | 2.41 ± 0.03 |
|  | 4.6 | 331.2 | 1.66 ± 0.05 |
|  | 4.7 | 329.2 | 1.66 ± 0.03 |
|  | 4.8 | 330.1 | 1.85 ± 0.00 |
|  | 4.9 | 323.0 | 1.70 ± 0.04 |
|  | 4.10 | 329.2 | 1.76 ± 0.07 |
|  | Average: | 329.5 ± 3.2 | 1.95 ± 0.27 |
|  |  |  |  |
| B5 | 5.1 | 161.2 | 0.57 ± 0.01 |
|  | 5.2 | 164.2 | 0.47 ± 0.02 |
|  | 5.3 | 162.1 | 0.39 ± 0.02 |
|  | 5.4 | 162.4 | 0.49 ± 0.02 |
|  | 5.5 | 161.9 | 0.28 ± 0.02 |
|  | 5.6 | 161.3 | 0.46 ± 0.01 |
|  | 5.7 | 161.1 | 0.46 ± 0.01 |
|  | 5.8 | 162.7 | 0.48 ± 0.02 |
|  | 5.9 | 163.4 | 0.54 ± 0.04 |
|  | 5.10 | 162.1 | 0.56 ± 0.01 |
|  | Average: | 162.2 ± 0.9 | 0.47 ± 0.08 |
|  |  |  |  |
| B6 | 6.1 | 122.5 | 0.51 ± 0.01 |
|  | 6.2 | 126.1 | 0.53 ± 0.02 |
|  | 6.3 | 134.2 | 0.59 ± 0.02 |
|  | 6.4 | 126.5 | 0.41 ± 0.01 |
|  | 6.5 | 125.7 | 0.44 ± 0.01 |
|  | 6.6 | 125.4 | 0.49 ± 0.01 |
|  | 6.7 | 126.8 | 0.52 ± 0.00 |
|  | 6.8 | 126.7 | 0.52 ± 0.02 |
|  | 6.9 | 124.2 | 0.51 ± 0.01 |
|  | 6.10 | 124.0 | 0.49 ± 0.01 |
|  | Average: | 126.2 ± 3.0 | 0.50 ± 0.05 |

**Table S3**. ^1^H and ^13^C NMR assignments of bromazolam. ^a^Chemical shifts (in ppm) were determined with reference to TMS. ^b^Spectra determined at 500 MHz. ^c^Spectra determined at 126 MHz. ^d,e^Equivalent environments with same chemical shift.

| Position | ^1^H Assignment^(a,b)^ | ^13^C Assignment^(c)^ |
| --- | --- | --- |
| 1 | - | 150.22 |
| 3a | - | 155.19 |
| 4 | - | 46.46 |
| 4a | 5.51 (d, *J* = 12.9 Hz, 1H) | - |
| 4b | 4.09 (d, *J* = 12.9 Hz, 1H) | - |
| 6 | - | 168.02 |
| 6a | - | 131.04 |
| 7 | 7.61 (d, *J* = 2.3 Hz, 1H) | 134.75 |
| 8 | - | 132.89 |
| 9 | 7.81 (dd, *J* = 8.6, 2.3 Hz, 1H) | 134.81 |
| 10 | 7.34 (d, *J* = 8.6 Hz, 1H) | 124.91 |
| 10a | - | 121.09 |
| 12 | 2.65 (s, 3H) | 12.46 |
| 1’ | - | 138.67 |
| 2’ | 7.57 – 7.51 (m)^d^ | 129.45^d^ |
| 3’ | 7.40 (dd, J = 8.3, 7.0 Hz)^e^ | 128.62^e^ |
| 4’ | 7.51 – 7.44 (m, 1H) | 131.10 |
| 5’ | 7.40 (dd, J = 8.3, 7.0 Hz)^e^ | 128.62^e^ |
| 6’ | 7.57 – 7.51 (m)^d^ | 129.45^d^ |

**Table S4.** GC–MS validation data for the quantification of bromazolam.

| **Parameter** | **Bromazolam** | | |
| --- | --- | --- | --- |
| SIM ions for quantification (base peak = **204**) | 325 | 352 | 353 |
| Relative intensities^a^ (%) | 68.2 | 35.9 | 15.2 |
| Precision (% RSD, n = 3) | 0.3 | 0.7 | 0.5 |
| t_R_ (min) | 12.48 | | |
| RRT^b^ | 1.68 | | |
| Rs^c^ | 20.5 | | |
| As^d^ | 1.45 | | |
| N^e^ (plates) | 917,055 | | |
| H^f^ (x 10^-5^ m) | 3.27 | | |
| Linearity (*r^2^*) | 0.999^g^ | | |
| LOD^h^ (mg/mL) | 3.35 | | |
| LOQ^i^ (mg/mL) | 10.2 | | |
| **Precision** (% RSD, n = 6) | | | |
| 20 mg/mL | 0.28 | | |
| 40 mg/mL | 0.36 | | |
| 60 mg/mL | 0.18 | | |
| 80 mg/mL | 0.18 | | |
| 100 mg/mL | 1.26 | | |
| 120 mg/mL | 0.61 | | |
| 140 mg/mL | 0.60 | | |
| **Assay Recovery** (%, n = 3) | | | |
| 80 mg/mL (80%) | 100.36 | | |
| 100 mg/mL (100%) | 98.13 | | |
| 120 mg/mL (120%) | 99.01 | | |
| Average Recovery (%) | 99.20 | | |
| Precision (% RSD) | 0.45 | | |
| Relative Error^j^ (%) | 0.80 | | |

*Key:* ^a^Relative intensities (compared to base peak) of GC-MS diagnostic ions used for bromazolam; ^b^Relative retention time (with respect to methyl stearate, t_R_ = 7.45 min); ^c^Resolution; ^d^Asymmetry (or tailing) factor; ^e^Number of theoretical plates; ^f^Height of a theoretical plate; ^g^y = 0.0089x + 0.0129; ^h^Limit of Detection (determined from the standard deviation of the response and slope of the calibration curve); ^i^Limit of Quantification (determined from the standard deviation of the response and slope of the calibration curve); ^j^Deviation between the average experimental recovery and a 100% recovery.

**Table S5**. Transition reactions monitored in LC-HR-MS/MS quantification of bromazolam.

| **Analyte** | **Ionization state** | **Precursor ion** | **Product ions** | **Retention time** |
| --- | --- | --- | --- | --- |
| Bromazolam | [M+H]^+^ | 353.0365 | 325.0214  274.1211 | 2.250 (+/- 0.2min) |
| Bromazolam-d5 | [M+H]^+^ | 358.0689 | 210.1073  279.1512 | 2.250 (+/- 0.2min) |

**Table S6.** Appearance of bromazolam tablets from 13 batches submitted to MANDRAKE for analysis.

| **Batch** | **Date of submission** | **Tablet appearance / presentation** |
| --- | --- | --- |
| M1 | 2022 | 1 x Green “XANAX” embossed bar |
| M2 | 2023 | 2 x White, Bensedin (10 mg) tablets in blister pack |
| M3 | 2023 | 10 x White, "XANAX" embossed bars |
| M4 | 2023 | 2 x White, circular tablets, no markings |
| M5 | 2023 | 10 x White, circular tablets, no markings |
| M6 | 2023 | 10 x Light blue, circular "MSJ" embossed tablets |
| M7 | 2023 | 5 x White, "XANAX" embossed bars |
| M8 | 2023 | 2 x Light blue, circular tablets no markings |
| M9 | 2023 | 2 x White, "XANAX" embossed bars |
| M10 | 2023 | 1 x Light blue, circular tablet no markings |
| M11 | 2024 | 1 x Light blue, circular "MSJ" embossed tablet |
| M12 | 2024 | 11 x Light blue, circular tablets no markings |
| M13 | 2024 | 2 x White, circular tablets, no markings |

**Table S7**. Comparison of ^1^H NMR Bromazolam quantification method using isotopic dilution LC-MS/MS analysis. LC-MS/MS quantitative analyses represent mean ± SD of three injections of the same sample (tablet extract). ^1^H NMR quantitative analyses represent three scans of same sample (tablet extract).

| Sample | Quantitative analysis (mg/tablet) | |
| --- | --- | --- |
|  | ^1^H NMR | LC-HR-MS/MS |
| B3 | 0.32 ± 0.02 | 0.32 ± 0.00 |
| B4.1 | 1.96 ± 0.06 | 2.07 ± 0.01 |
| B5.1 | 0.57 ± 0.01 | 0.53 ± 0.00 |
| B6.1 | 0.51 ± 0.01 | 0.45 ± 0.00 |
| B7 | 0.09 ± 0.00 | 0.08 ± 0.00 |

**Table S8**. Transition reactions monitored in LC-HR-MS/MS detection of cocaine from sample B7.

| **Analyte** | **Ionization state** | **Precursor ion** | **Product ions** | **Retention time** |
| --- | --- | --- | --- | --- |
| Cocaine | [M+H]^+^ | 304.1546 | 182.1175,  82.0651,  105.0335 | 2.383 |

**Figure S1**. IR spectrum of bromazolam sample B1 (tablet extract in CHCl_3_) measured in liquid cell. IR vmax (KBr, cm^-1^) 3401, 2923 Ar(C-H) stretch, 1758 Ar(C=N) stretch, 1485 Ar(C=C) stretch, 1184 (C-N) stretch, 733 Ar(C-H) bend, 698 (C-Br) stretch.


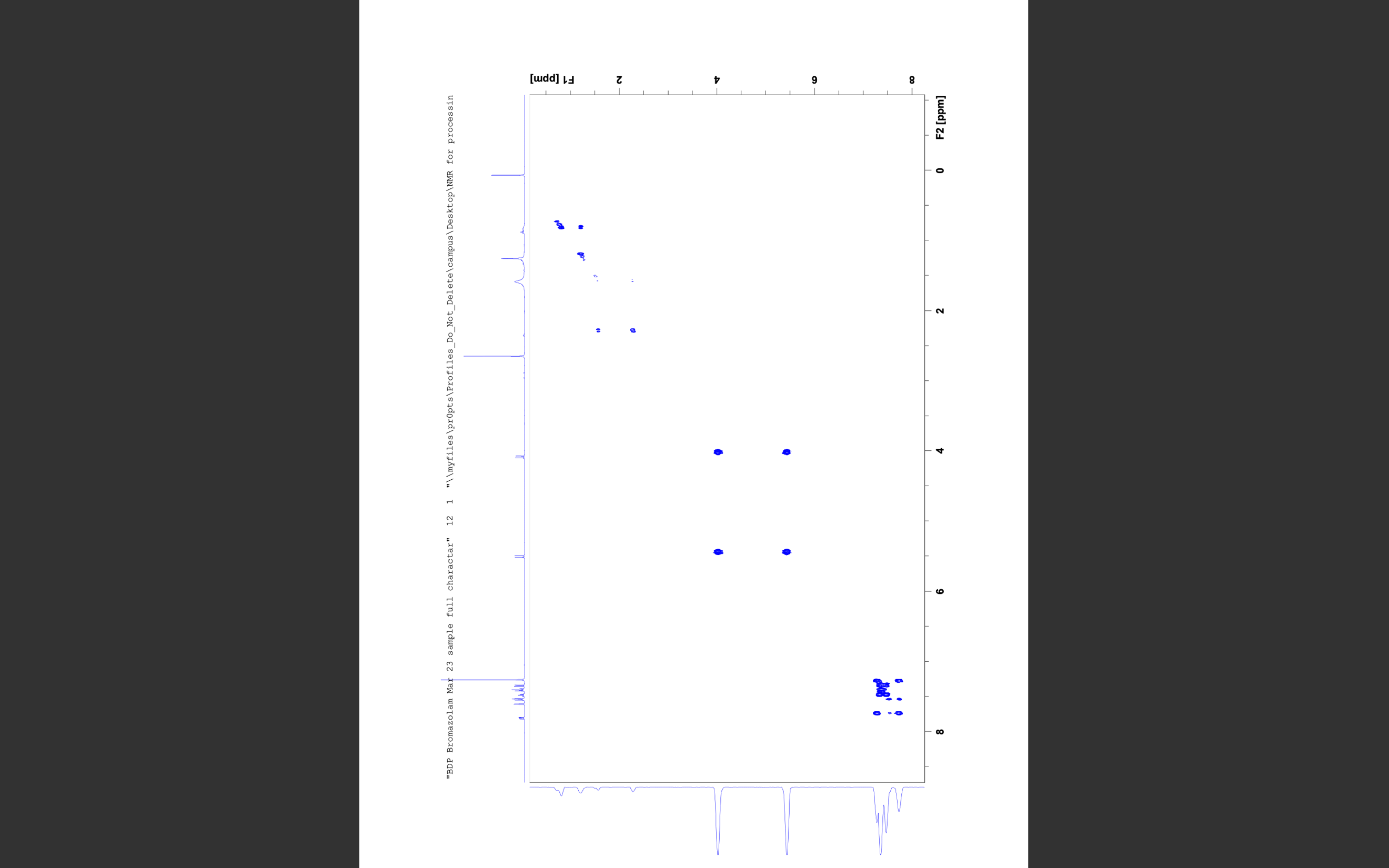


**Figure S2**. Homonuclear Correlation Spectroscopy (COSY) analysis of bromazolam tablet sample B1. Acquired in CDCl_3_.


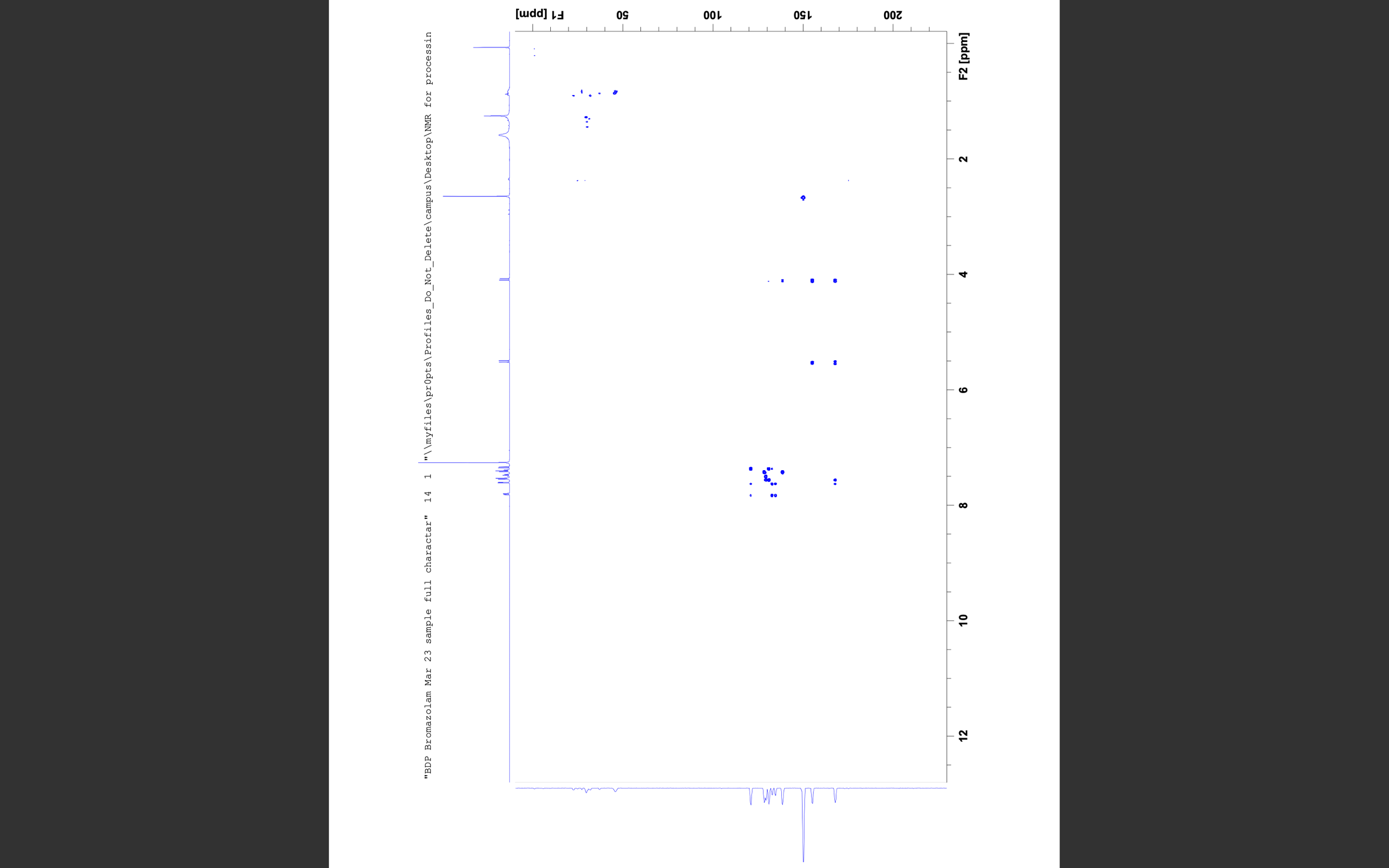


**Figure S3**. Heteronuclear Multiple Bond Correlation (HMBC) analysis of bromazolam tablet sample B1. Acquired in CDCl_3_.


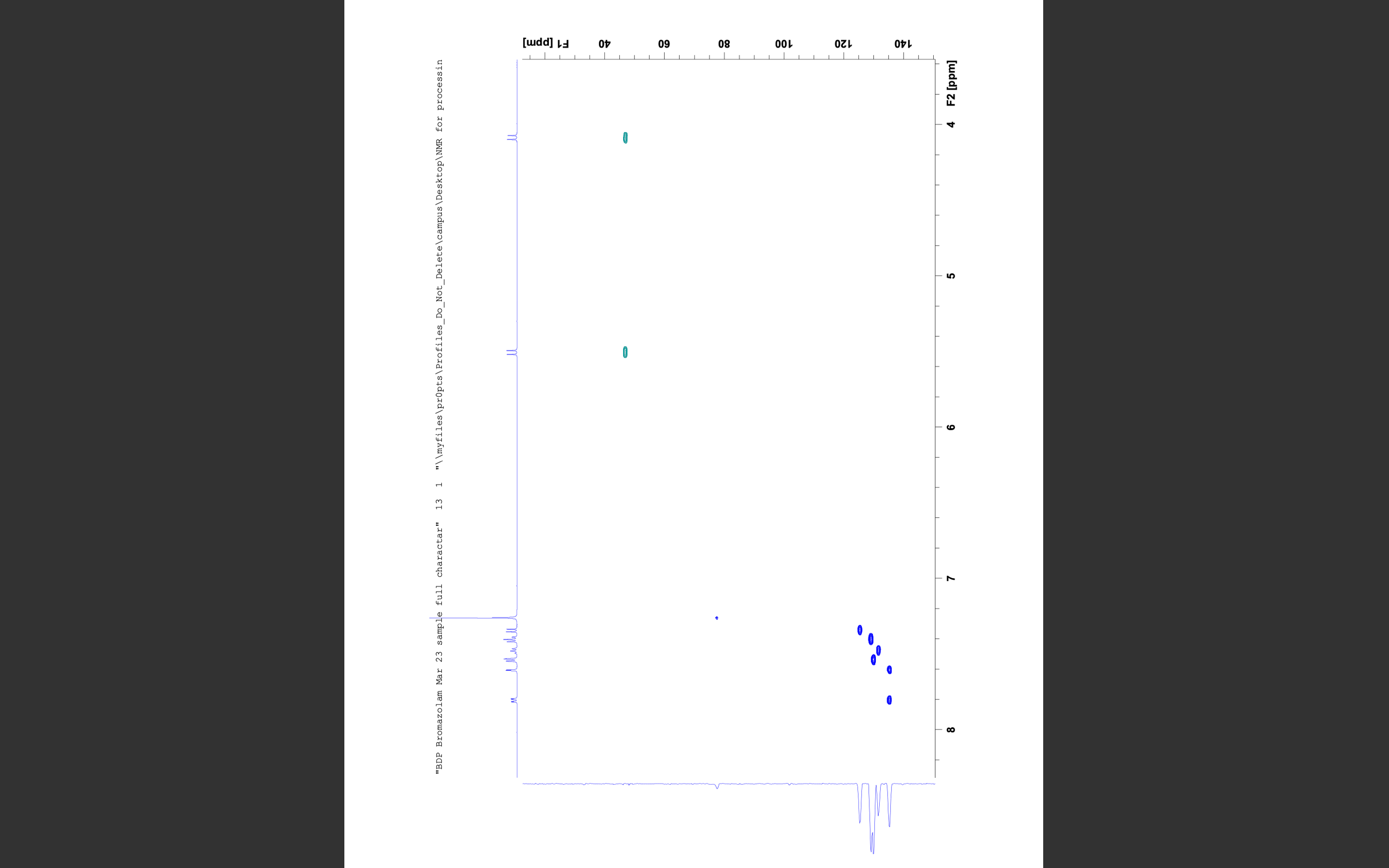


**Figure S4.** Heteronuclear Single Quantum Coherence (HSQC) spectrum of bromazolam tablet extract from sample B1. Acquired in CDCl_3_.


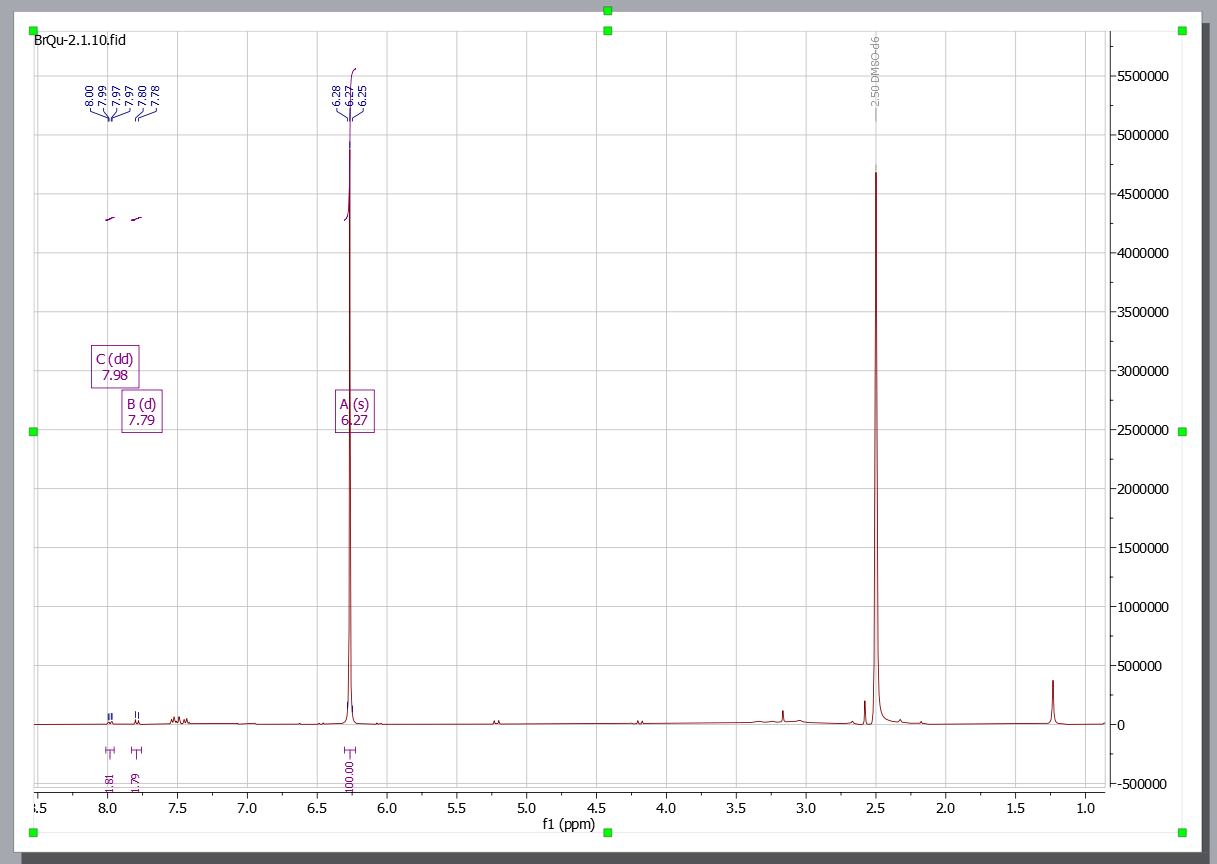


**Figure S5**. Exemplar ^1^H NMR spectrum of bromazolam tablet extract B4.1. Ran in deuterated DMSO-D6 (singlet peak at 2.5 ppm). Maleic acid used as internal standard showing singlet peak at 6.27 ppm. H7 and H9 integrals of bromazolam used in quantification are shown at 7.79 ppm, doublet (d) and 7.98 ppm, double of doublets (dd).

**
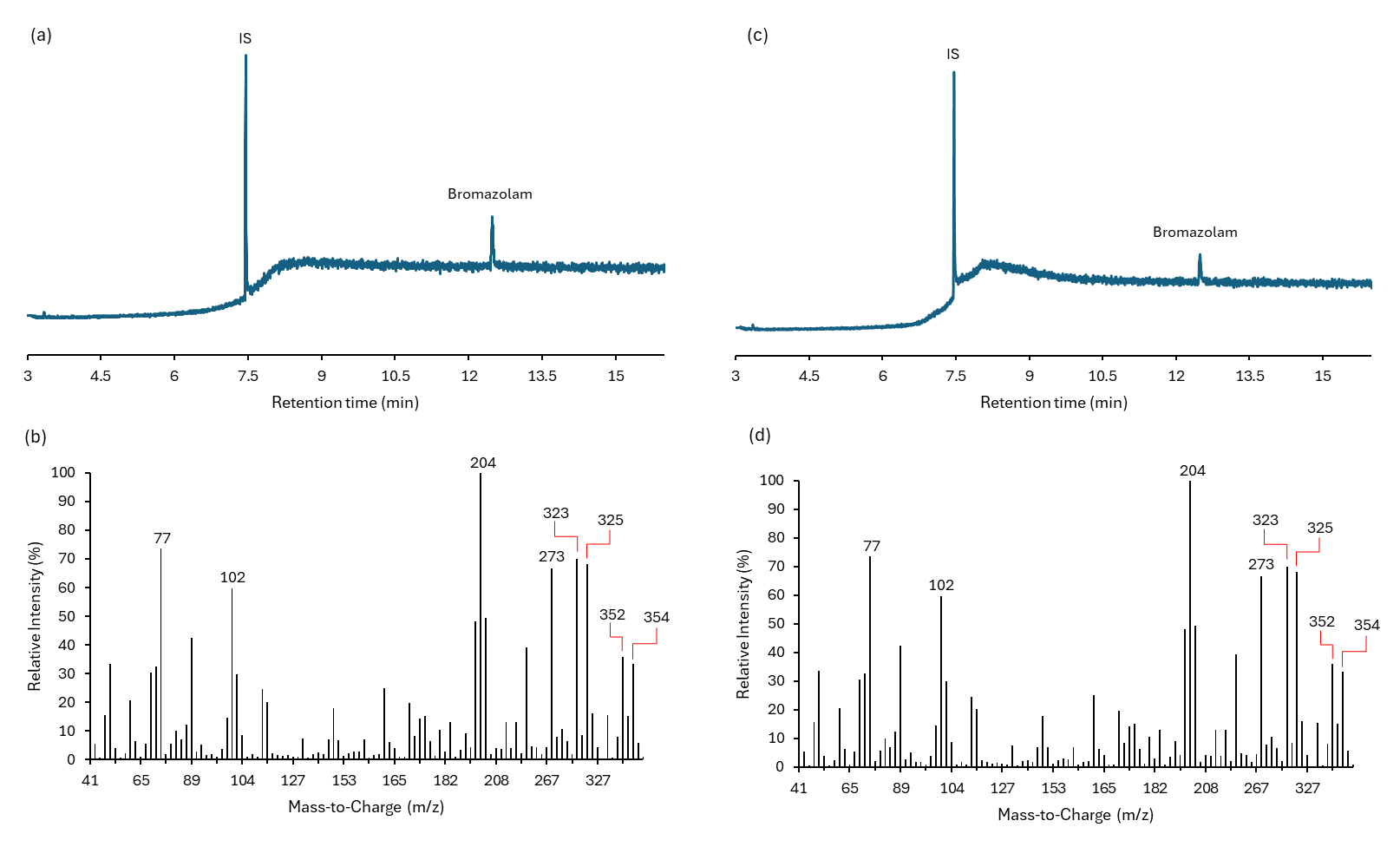
**

**Figure S6.** (a) Representative total ion chromatogram of bromazolam (1 mg/mL) containing methyl stearate (IS, 70 μg/mL) in methanol; (b) EI-MS spectrum (+ve ion mode) of bromazolam (t_R_ = 12.48 minutes) standard; (c) representative total ion chromatogram of Green “XANAX” embossed bar (Sample M1) containing methyl stearate (IS, 70 μg/mL); (d) EI-MS spectrum (+ve ion mode) of Sample M1 (t_R_ = 12.48 minutes).


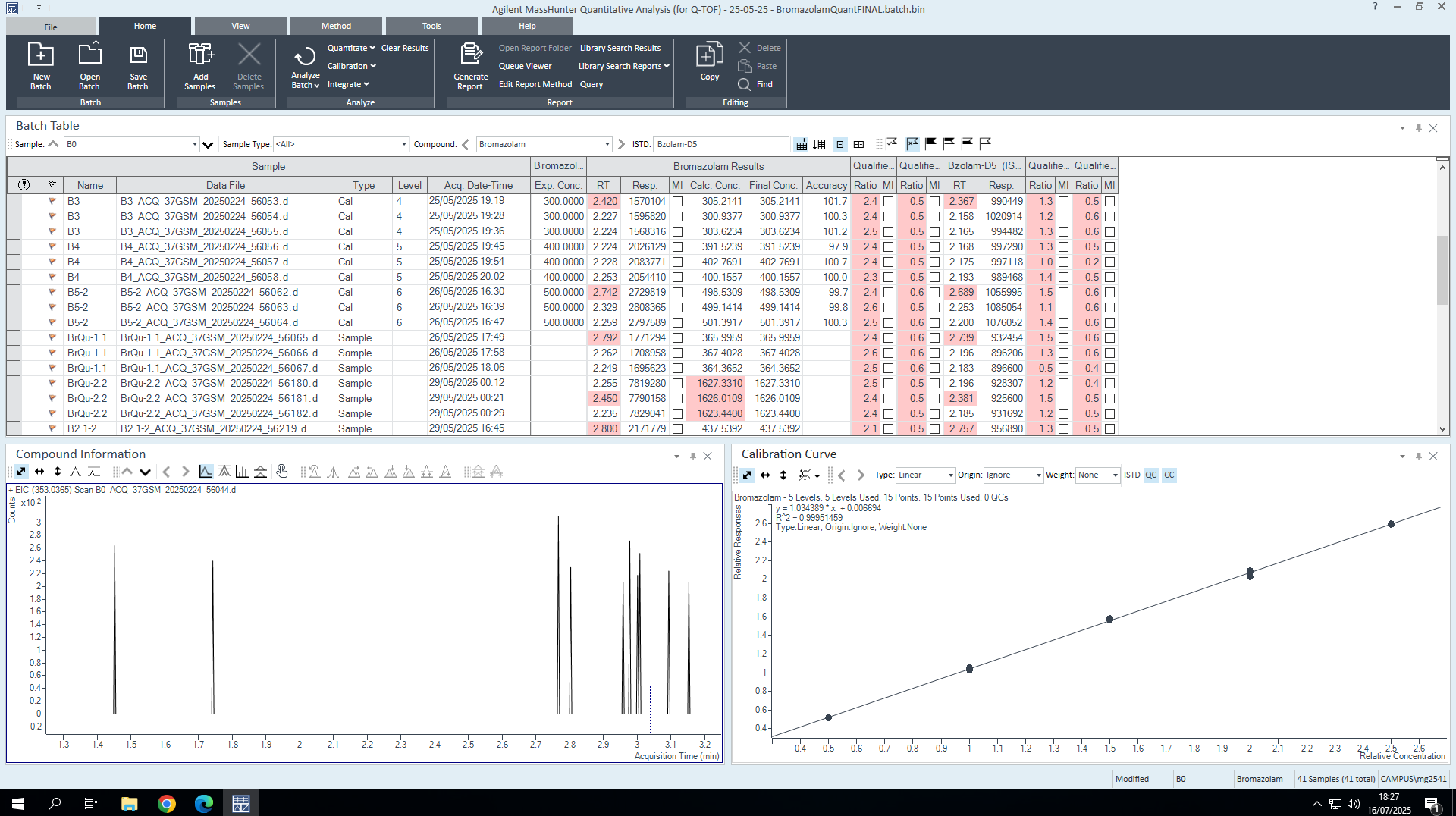


**Figure S7**. LC-HR-MS/MS isotopic dilution calibration curve for bromazolam/bromazoalm-d5


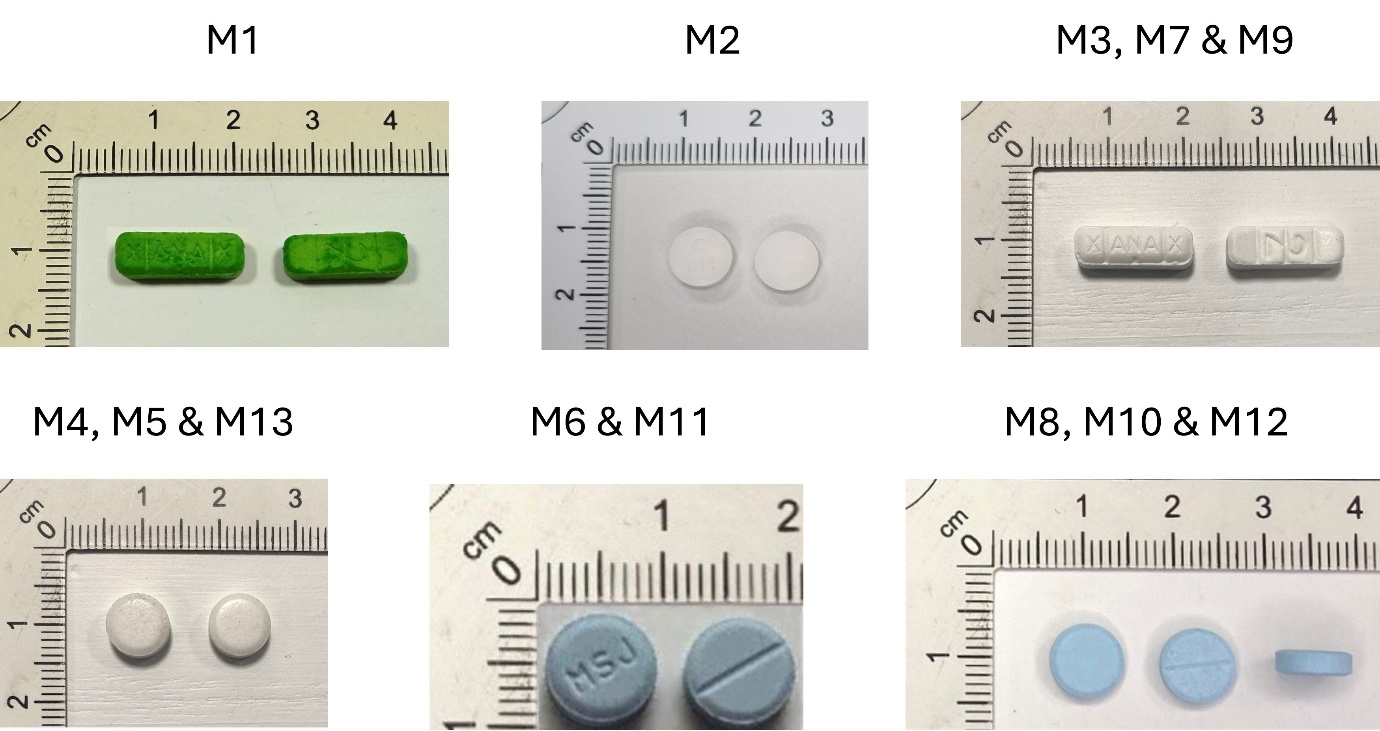


**Figure S8.** Appearance of bromazolam tablets from batches M1-M13 submitted to MANDRAKE 2022-2025.

**Figure S9.** Total number of deaths with bromazolam or other NPS benzodiazepine detections reported to the NPSUM by 1^st^ November 2024. Note: Due to the time taken between a death occurring and conclusion of coronial inquest, which is when cases are reported to the NPSUM, it is anticipated that further cases will be reported where death occurred 2022-2024. The projected number of deaths anticipated to be received has been calculated using previous jurisdiction reporting trends.
